# AhR activation attenuates calcium oxalate nephrocalcinosis by diminishing M1 macrophage polarization and promoting M2 macrophage polarization

**DOI:** 10.7150/thno.51144

**Published:** 2020-10-25

**Authors:** Xiaoqi Yang, Haoran Liu, Tao Ye, Chen Duan, Peng Lv, Xiaoliang Wu, Jianhe Liu, Kehua Jiang, Hongyan Lu, Huan Yang, Ding Xia, Ejun Peng, Zhiqiang Chen, Kun Tang, Zhangqun Ye

**Affiliations:** 1 Department of Urology, Tongji Hospital, Tongji Medical College, Huazhong University of Science and Technology, Wuhan, China.; 2 Department of Urology, The Second Affiliated Hospital of Kunming Medical University, Kunming, China.; 3 Department of Urology, Guizhou Provincial People's Hospital, Guiyang, China.; 4 Department of Urology, The Third Affiliated Hospital of Chongqing Medical University, Chongqing, China.

**Keywords:** AhR, IRF1, HIF-1α, Macrophage, Nephrocalcinosis

## Abstract

Calcium oxalate (CaOx) crystal can trigger kidney injury, which contributes to the pathogenesis of nephrocalcinosis. The phenotypes of infiltrating macrophage may impact CaOx-mediated kidney inflammatory injury as well as crystal deposition. How aryl hydrocarbon receptor (AhR) regulates inflammation and macrophage polarization is well understood; however, how it modulates CaOx nephrocalcinosis remains unclear.

**Methods:** Mice were intraperitoneally injected with glyoxylate to establish CaOx nephrocalcinosis model with or without the treatment of AhR activator 6-formylindolo(3,2-b)carbazole (FICZ). Positron emission tomography computed tomography (PET-CT) imaging, Periodic acid-Schiff (PAS) staining, and polarized light optical microscopy were used to evaluate kidney injury and crystal deposition in mice kidney. Western blotting, immunofluorescence, chromatin immunoprecipitation, microRNA-fluorescence *in situ* hybridization, and luciferase reporter assays were applied to analyze polarization state and regulation mechanism of macrophage.

**Results:** AhR expression was significantly upregulated and negatively correlated with interferon-regulatory factor 1 (IRF1) and hypoxia inducible factor 1-alpha (HIF-1α) levels in a murine CaOx nephrocalcinosis model following administration of FICZ. Moreover, AhR activation suppressed IRF1 and HIF-1α levels and decreased M1 macrophage polarization *in vitro*. In terms of the mechanism, bioinformatics analysis and chromatin immunoprecipitation assay confirmed that AhR could bind to miR-142a promoter to transcriptionally activate miR-142a. In addition, luciferase reporter assays validated that miR-142a inhibited IRF1 and HIF-1α expression by directly targeting their 3'-untranslated regions.

**Conclusions:** Our results indicated that AhR activation could diminish M1 macrophage polarization and promote M2 macrophage polarization to suppress CaOx nephrocalcinosis via the AhR-miR-142a-IRF1/HIF-1α pathway.

## Introduction

Kidney stones are one of the most common urological disorders, and their incidence and prevalence are increasing worldwide 1, 2. Calcium oxalate (CaOx) stones comprise approximately 80% of kidney stones and can cause nephrocalcinosis 3. CaOx crystals trigger renal tubular cell injury, which contributes to the pathogenesis of nephrocalcinosis 4, 5.

Macrophages with a major feature of plasticity participate in first-line defense against pathogens, homeostatic maintenance and inflammatory responses 6. Classically activated macrophages (M1 macrophages) and alternatively activated macrophages (M2 macrophages) are two subtypes of macrophages 7, 8. M1 macrophages have been shown to promote inflammation-associated oxidative stress to drive the deposition of crystal, whereas M2 macrophages eliminate crystal deposition through phagocytosis 9-13.

For many decades, homeostasis of the immune and inflammatory systems was considered to be critical for the prevention and treatment of urinary stones. Aryl hydrocarbon receptor (AhR) is a ligand-activated transcription factor involved in a series of crucial pathological processes, especially immune and inflammatory responses 14, 15. It is worth noting that Butlera et al. found several 3- to 4-mm uric acid stones in the bladder of 6-month-old AhR knockout mouse 16. In addition, AhR is important for the dynamic phenotypic balance between pro-inflammatory M1 macrophages and anti-inflammatory M2 macrophages 17. However, the roles of the AhR signalling pathway in controlling infiltrating macrophages to impact intrarenal CaOx crystal deposition and elimination remain largely unknown.

Herein, we assessed the impact of AhR activation on CaOx nephrocalcinosis, revealing that the AhR-miR-142a-IRF1/HIF-1α axis attenuates CaOx nephrocalcinosis-mediated kidney injury and crystal deposition by diminishing M1 macrophage polarization and promoting M2 macrophage polarization, and highlighting the novel technology of ^18^F-FDG PET-CT, which provides anatomical and metabolic information to define the extent of renal inflammatory status and assess response to treatment. AhR may offer value as a therapeutic target for treating or preventing CaOx nephrocalcinosis.

## Methods

### Animal experiment

Male C57BL/6J mice (6-8 weeks) were obtained from the Experimental Animal Research Center of Hubei and were housed under pathogen-free conditions at Tongji Hospital. The “NIH Guide for the Care and Use of Laboratory Animals" was used to guide animal care, and all animals were randomly allocated to different experimental and control groups. To establish a murine CaOx nephrocalcinosis model, each mouse was intraperitoneally injected with glyoxylate (Gly) (75 mg/kg/d) or saline from day 4 to day 10 18. Animals were euthanized and underwent further laparotomy and evaluation. A murine CaOx nephrocalcinosis model was successfully established to better study whether crystal deposition was observed by polarized light optical microscopy. Animals in the intervention groups were intraperitoneally injected with increasing concentrations (50, 100, 200 mg/kg/d) of 6-formylindolo(3,2-b)carbazole (FICZ) from day 1 to day 10. Mice received tail vein injections with antagomiR-142a (RiboBiotech, Guangzhou, China) (20 mg/kg, 200 µl) on days 1, 4, and 7. The study was approved by the Ethics Committee of Tongji Hospital, Tongji Medical College, Huazhong University of Science and Technology.

### Positron emission tomography computed tomography (PET-CT) imaging

^18^F-fluorodeoxyglucose (^18^F-FDG) is a deoxyglucose analogue that accumulates at higher levels in tissues that utilize greater amounts of glucose. Activated inflammatory cells that consume a large amount of glucose similarly exhibit high ^18^F-FDG uptake 19. Recently, an increasing number of studies have used ^18^F-FDG PET-CT to assess renal inflammation and acute renal injury 20-22. Herein, we retrospectively studied 628 patients with malignant disease who underwent delayed-imaging PET-CT at Tongji Hospital from January 2016 - January 2018. Kidney stones were found in sixty-two patients by PET-CT scans. ^18^F-FDG uptake was used to evaluate the inflammation state of the renal parenchyma around the stone. Residual urine radionuclide signals were eliminated with a GE threshold segmentation model that was utilized as a means of removing the renal pelvis 13. Standardized uptake values (SUVs) were determined for the region of interest (ROI).

For the *in vivo* experiment, each animal received a caudal vein injection of 200 ± 10 μCi ^18^F-FDG. One hour later, each mouse was anaesthetized with 2% isoflurane, and then a static PET scan was conducted for 10 minutes using a Trans-PET BioCaliburn 700 System (Raycan Technology Co., Ltd., Suzhou, China). Then, the 3D ordered subset expectation-maximization method (voxel size: 0.5×0.5×0.5 mm^3^) was used to reconstruct the data.

### Measurement of serum creatinine and blood urea nitrogen (BUN) levels

Murine blood samples were collected on days 3 and 10, after which commercially purchased kits (Stanbio Laboratory, TX, USA) were used to measure serum BUN and creatinine levels.

### Renal CaOx crystal detection

Mouse renal tissue was stained with haematoxylin-eosin following a routine experimental procedure and then visualized via polarized light optical microscopy (Zeiss, Germany). In addition, the Pizzolato staining method can also be used to evaluate crystal deposition 23.

### Periodic acid-Schiff (PAS) staining

Kidney tissue sections were stained with PAS to assess tubular injury, which includes tubular dilation, cast formation, tubular cell sloughing, atrophy, and basement membrane thickening. The overall frequency of injured tubular epithelial cells was determined and scored as follows: none, 0; <25%, 1; 25-50%, 2; 51-75%, 3; and >75%, 4. Average scores in 10 fields examined were then calculated to assess the average degree of injury 24.

### Terminal deoxynucleotidyl transferase dUTP nick end labeling (TUNEL) staining

TUNEL staining was conducted using an *In situ* Cell Death Detection Kit (Roche, Rotkreuz, Switzerland) following the manufacturers' instructions. TUNEL-positive cells were quantified in ten randomly chosen fields from each section to evaluate renal cell necrosis 25.

### Immunohistochemistry (IHC)

For IHC, mouse kidney slices were incubated with anti-AhR (1:100, Boster, Wuhan, China), anti-HIF-1α (1:100, Boster), or anti-IRF1 (1:1000, Absin, Shanghai, China) antibodies overnight at 4°C. The antibodies were detected with an Envision HRP Polymer System (Boster), and the slices were scanned with a Leica SCN400 scanner.

### MicroRNA-fluorescence *in situ* hybridization (FISH)

An RB200-labelled mmu-miR-142a-3p probe (RiboBiotech) and a FISH kit (RiboBiotech) were used for these analyses. Images were captured by a rapid scanning fluorescence microscope (Olympus, Tokyo, Japan).

### Cells and Chemicals

Renal tubular epithelial cells (TECs) were extracted from renal cortex of C57BL/6J mice. In brief, fragmented renal cortex was dissociated with 1 mg/mL type I collagenase for 0.5 hour at 37°C, then isolated by gradient-density centrifugation in Percoll. Bone marrow derived macrophages (BMDMs) were extracted from the femurs and tibias of C57BL/6J mice and RPMI 1640 supplemented with 10% fetal bovine serum in a humidified atmosphere of 5% CO2. BMDMs were treated with macrophage colony-stimulating factor (M-CSF) (BD Biosciences, Franklin Lakes, NJ, USA) at concentration of 50 ng/mL for 7 days. After discarding non-adherent cells on day 5, remaining cells were used for subsequent experiments. TECs were exposed to 100 μg/mL calcium oxalate monohydrate (COM) for 24 hours and co-cultured with BMDMs in lower and upper chamber of Transwell plates (Corning, Inc., Corning, NY, USA) to simulate microenvironment of macrophages in mouse kidney.

FICZ was obtained from MedChem Express (USA). Four miRNA oligonucleotides (miR-142a mimic, miR-142a inhibitor, and corresponding negative controls) were provided by RiboBiotech (Guangzhou, China). A siRNA targeting AhR was obtained from RiboBiotech (Guangzhou, China). The siAhR sequence was 5′-TTAGGGUGUAGGCGUACUAAU-3′. These miRNA oligonucleotides and AhR siRNA were transfected into cells using riboFECTTM CP according to the provided instructions (RiboBiotech).

### Real-time quantitative reverse transcriptase-polymerase chain reaction (qRT-PCR)

TRIzol (Invitrogen, CA, USA) was employed to extract total RNA from BMDMs or frozen mouse kidney. Subcellular fractionation of nuclear RNA was performed by using Norgen's Cytoplasmic and nuclear RNA purification kit (Norgen BioTek, Canada) according to the manufacturer's protocol. Then PrimeScript RT Kit (TaKaRa, Shiga, Japan) was utilized for cDNA preparation, and SYBR Green Master Mix (Yeasen, Shanghai, China) was used for qPCR based on standard protocols. β-actin was used for the normalization of relative gene expression. The murine primers are shown in Table S1. In addition, mature miRNAs were assessed by the All-in-One miRNA qRT-PCR Detection Kit (GeneCopoeia, MD, USA) following the manufacturer's protocols, with U6 RNA being used for normalization.

### Western blotting

Total and nuclear proteins were extracted from BMDMs in RIPA buffer containing protease inhibitors (Servicebio, Wuhan, China). Then protein extracts were separated by 10% SDS-PAGE and incubated overnight at 4°C with primary antibodies specific for AhR (BA2013, 96 kDa, 1:200, Boster, China), HIF-1α (PB9253, 97 kDa, 1:1,000, Boster, China), IRF1 (abs118047, 37 kDa, 1:1,000, Absin, China), NF-κB p65 (GB11142-1, 65 kDa, 1:1,000, Servicebio, China), iNOS (BA0362, 130 kDa, 1:200, Boster, China), Arg-1 (GB11285, 40 kDa, 1:5,000, Servicebio, China), H3 (GB13488, 15 kDa, 1:1,000, Servicebio, China), and β-actin (BA2305, 43 kDa, 1:1,000, Boster, China). Blottings were then probed with appropriate secondary antibodies for 2 h at 25°C and assessed via an ECL kit (Millipore, MA, USA). Triplicate analyses of all samples were conducted.

### Immunofluorescence

Paraformaldehyde was used to fix BMDMs on glass slides, after which they were blocked using goat serum prior to probing overnight at 4°C with anti-Arg-1 (16001-1-AP; 1:200; Proteintech Group, Wuhan, China) or anti-iNOS (610431; 1:200; BD Biosciences). Samples were then stained using secondary antibodies labelled with Cy3 or AF488 (1:1000; Thermo Fisher Scientific), followed by DAPI staining and mounting. Samples were assessed via fluorescence microscopy (Nikon, Tokyo, Japan), with all samples being assessed with the same settings.

Deparaffinization and antigen retrieval were employed to prepare murine paraffin-embedded renal tissue sections, which were then treated with goat serum as a blocking reagent prior to overnight incubation at 4°C with anti-Arg-1 (sc-271430; 1:200; Santa Cruz Biotechnology, TX, USA) or anti-iNOS (BA0362, 1:200, Boster). Samples were then probed with secondary antibodies conjugated to FITC or Cy3 (1:1000; Thermo Fisher Scientific), followed by fluorescence microscopic evaluation as above.

### Quantification of cytokine levels

Enzyme-linked immunosorbent assay (ELISA) kits (R&D Systems, Minneapolis, MN, USA) were used to measure the levels of IL-1β, TNF-α, IL-6, and IL-10 in supernatant samples. Murine serum cytokines were measured based on the provided ELISA kit instructions.

### Internalization of COM crystal by BMDMs

To reveal the interaction between COM and BMDMs, COM crystal (1 mg/mL) were incubated with Alexa Fluor-488-conjugated IgG (4412S; 1:400; CST, USA) at 0.01 mg/mL for 3 h in the dark at 25℃. We first co-cultured COM-stimulated TECs with treated BMDMs for 24 h. Next, BMDMs were cultured for 5 h in the presence of 100 µg/mL fluorescent COM crystal. The internalized crystal was then observed under a Nikon fluorescence microscope (Nikon).

### Chromatin immunoprecipitation (ChIP)

The EZ-Magna ChIP Assay Kit (Millipore) was employed to analyse the region of the miR-142a-3p promoter directly bound by AhR. BMDMs were fixed with 1% formaldehyde for 10 min at 37°C. Subsequently, the crosslinking reaction was terminated with 0.125 M glycine, and the BMDMs were washed twice with PBS and centrifuged (800 × g, 4°C, 5 min). Following cellular lysis, the crosslinked chromatin was sheared into 200-1000 bp fragments via sonication. Anti-AhR (MA1-513; 1:100; ThermoFisher) was then used to immunoprecipitate samples, and DNA was purified with magnetic beads prior to amplification via ChIP-qPCR using a primer pair (F: 5′-TTGCGAGAGTGGGAGAGT-3′, R: 5′-CCTCATTCCTGCTCCTCAA-3′).

### Luciferase reporter assay

Luciferase reporter vectors were purchased from Vigene Biosciences (Shandong, China). For reporter plasmid construction, the 3′-untranslated regions (3′-UTRs) of the target genes (IRF1 and HIF-1α), which harbour miR-142a-3p binding sites, and mutated sites (IRF1 mutated from ACACUA to UCUCAA; HIF-1α mutated from AACACUA to AUCAGUU) were cloned into the psiCHECK2 vector. BMDMs were co-transfected with miR-142a-3p mimic and the corresponding plasmid. The Dual-Luciferase Assay System was utilized to evaluate luciferase activity at 24 h posttransfection.

### Statistical analysis

GraphPad Prism (v.8.0.1; GraphPad Software, CA, USA) was utilized to analyse the data. Data are means ± SD. Two-tailed Student's t tests and one-way ANOVAs were performed to analyse group differences. Pearson's correlation test was performed to analyse correlations between two genes. A *P*-value < 0.05 was the significance threshold.

## Results

### High inflammation status and AhR dysregulation in kidney stone patients

**Figure 1:**
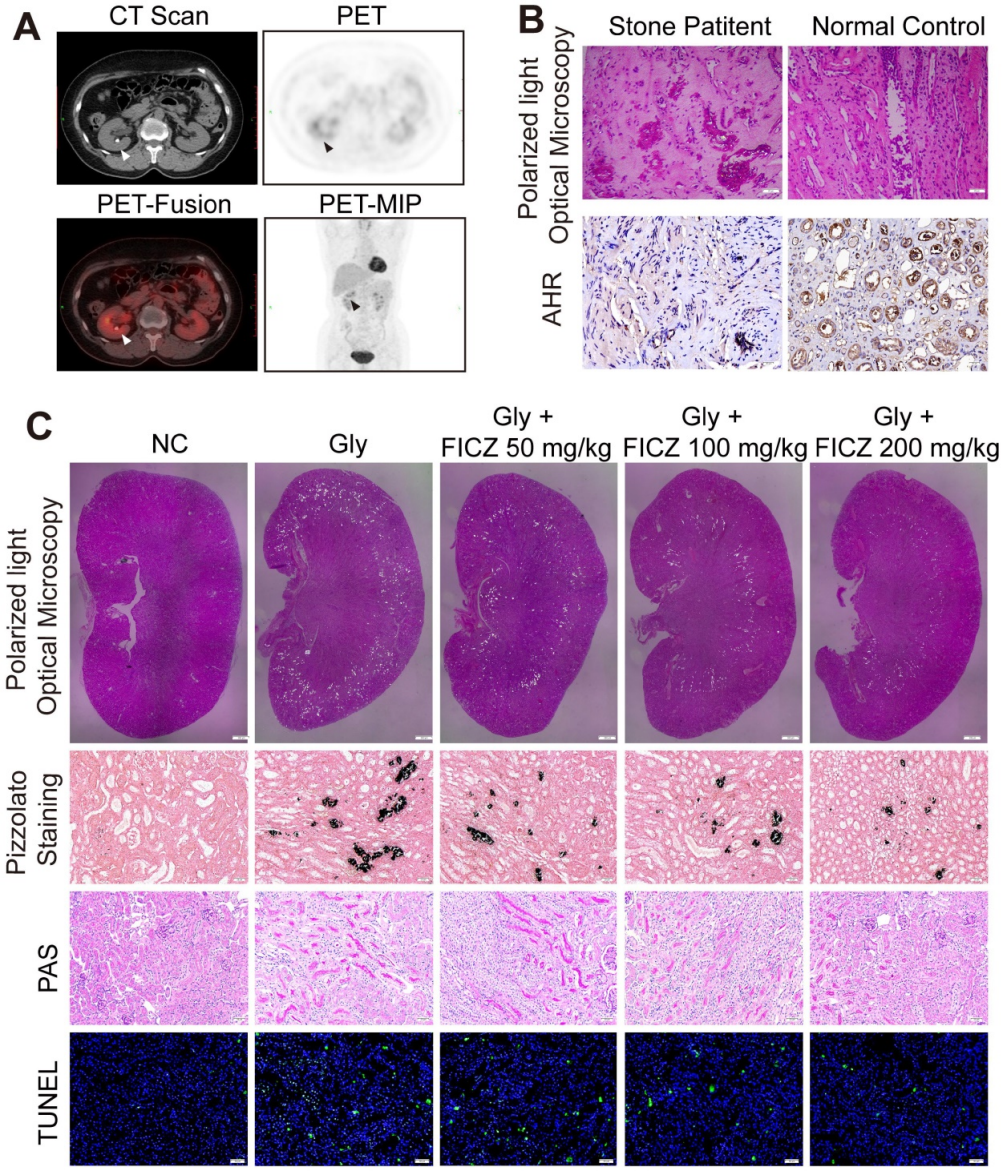
** High inflammation status and AhR dysregulation in stone patient kidneys while reducing renal inflammation and injury found in AhR-activated CaOx nephrocalcinosis mice.** (A) PET-CT imaging studies assessing renal inflammatory responsiveness. Axial CT, axial PET, axial fused PET-CT and coronal PET maximum intensity projection (MIP) images suggesting enhanced renal uptake of ^18^F-FDG. Arrows are used to mark focal ^18^F-FDG accumulation in the form of a ring surrounding the stone. (B) crystal deposition in Randall's plaques (n = 10) was analysed via polarized light optical microscopy (100×; scale bar: 20 µm) and IHC staining for AhR in Randall's plaques (200×; scale bar: 20 µm). (C) Deposition of renal CaOx crystal in the corticomedullary junction of mice (n = 6) treated with increasing concentrations of FICZ was analysed via polarized light optical microscopy (20×, scale bar: 500 µm). Crystal deposition within corticomedullary junction regions was further confirmed by Pizzolato staining (200×; scale bar: 20 µm). Kidney injury and necrosis were evaluated by PAS staining (200×; scale bar: 20 µm) and TUNEL staining (200×; scale bar: 50 µm) in kidney tissues, respectively.

We retrospectively studied 628 patients with malignant disease who underwent delayed-imaging PET-CT at Tongji Hospital between January 2016 and January 2018. A total of 62 of the patients were found to have kidney stones on scan. As shown in Figure 1A and Figure S1A, we found that the uptake of ^18^F-FDG was significantly higher in the renal parenchyma of the stone side than in the healthy side of the same patient (*p*<0.01). This phenomenon reflects the high degree of inflammation of the renal parenchyma caused by kidney stones. Randall's plaques were also observed in stone patients who underwent percutaneous nephrolithotomy by polarized light optical microscopy and IHC. Significant CaOx crystal deposition with low AhR expression was found in stone patients (Figure 1B, Figure S1B-C).

### *In vivo* activation of AhR suppresses the deposition of CaOx crystal and nephrocalcinosis-mediated renal inflammation and injury

To explore how AhR activation protects against the renal deposition of CaOx crystal and CaOx nephrocalcinosis-mediated kidney injury, mice were administered the AhR agonist FICZ for 3 days at an increasing dose, after which a model of CaOx nephrocalcinosis was established for 7 days (along with FICZ treatment). Consistent with polarized light optical microscopy, Pizzolato staining showed that CaOx crystal deposition significantly decreased as the FICZ concentration increased. Further TUNEL and PAS staining revealed the dose-dependent inhibition of nephrocalcinosis-mediated renal inflammatory damage upon FICZ treatment (Figure 1C, Figure S1D-G).

### AhR significantly suppressed IRF1 and HIF-1α expression in a murine model of CaOx nephrocalcinosis

**Figure 2:**
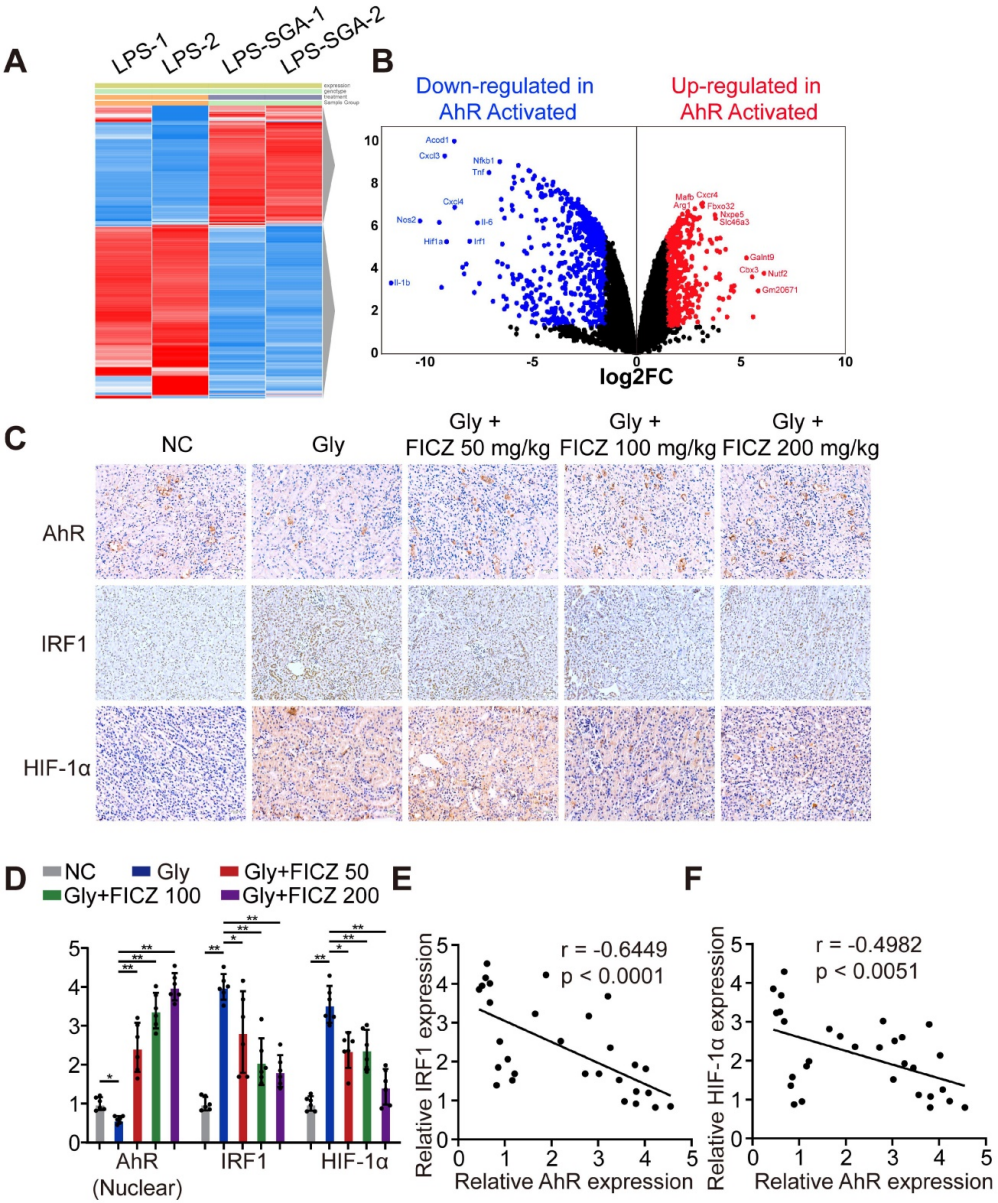
** AhR significantly suppressed IRF1 and HIF-1α expression in a murine CaOx nephrocalcinosis model.** (A) RNA-seq heatmap showing significantly altered mRNAs in SGA-treated BMDMs. (B) Volcano plots showing mRNA transcripts that were differentially expressed between LPS-treated and SGA-treated BMDMs. Significantly downregulated and upregulated mRNAs are shown in green and red, respectively, whereas genes that were not significantly changed are shown in black. (C) IHC staining for AhR, IRF1, and HIF-1α in the kidneys of FICZ-treated mice with CaOx nephrocalcinosis (200×; scale bar: 20 µm). (D) qRT-PCR was used to assess AhR, IRF1, and HIF-1α expression in kidney samples from FICZ-treated mice (n = 6) with CaOx nephrocalcinosis compared to kidney samples from model mice. (E, F) Pearson's correlation coefficient analysis (n = 30) of the expression levels of AhR and IRF1 (E) or HIF-1α (F). Each dot represents an individual animal. *P < 0.05; **P < 0.01, as assessed via one-way ANOVA (D).

To discover key genes regulated by AhR in BMDMs after inflammatory stimulation, we searched the GEO database and found an available dataset, GSE98810, analysing the gene expression of AhR in stimulated inflammatory macrophages. RNA sequencing (RNA-seq) showed that AhR activation in BMDMs caused the upregulation of 201 genes and the downregulation of 579 genes (|Log_2_FC| ≥ 1; P-value < 0.05) (Figure 2A-B). Gene Ontology (GO) was used to further analyse the most significant functions and processes associated with these differentially expressed genes. “Cytokine-mediated pathway,” “inflammatory response,” “type I interferon pathway”, “macrophage phagocytosis pathway”, and “NF-κB pathway” were ranked in the top 15 significant GO pathways (Figure S2). These results led us to hypothesize that IRF1 and HIF-1α may impact inflammatory diseases through AhR-related regulatory mechanisms. We then tested this by treating mice with glyoxylate-induced renal CaOx nephrocalcinosis using rising FICZ doses. Increased IHC staining for AhR and decreased staining for IRF1 and HIF-1α (which control M1 macrophage polarization) were observed in an FICZ-dependent manner (Figure 2C, Figure S3A-C). We also observed reductions in IRF1 and HIF-1α levels, while AhR expression was increased (Figure 2D). Next, we examined the relationships between AhR, IRF1 and HIF-1α expression using Pearson's correlation coefficient analysis and found that AhR expression was negatively correlated with IRF1 and HIF-1α expression (Figure 2E, F). These findings showed that FICZ treatment substantially increased AhR, which could decrease IRF1 and HIF-1α expression in a murine CaOx nephrocalcinosis model.

### AhR suppressed IRF1 and HIF-1α to attenuate CaOx crystal-stimulated M1 macrophage polarization *in vitro*

**Figure 3:**
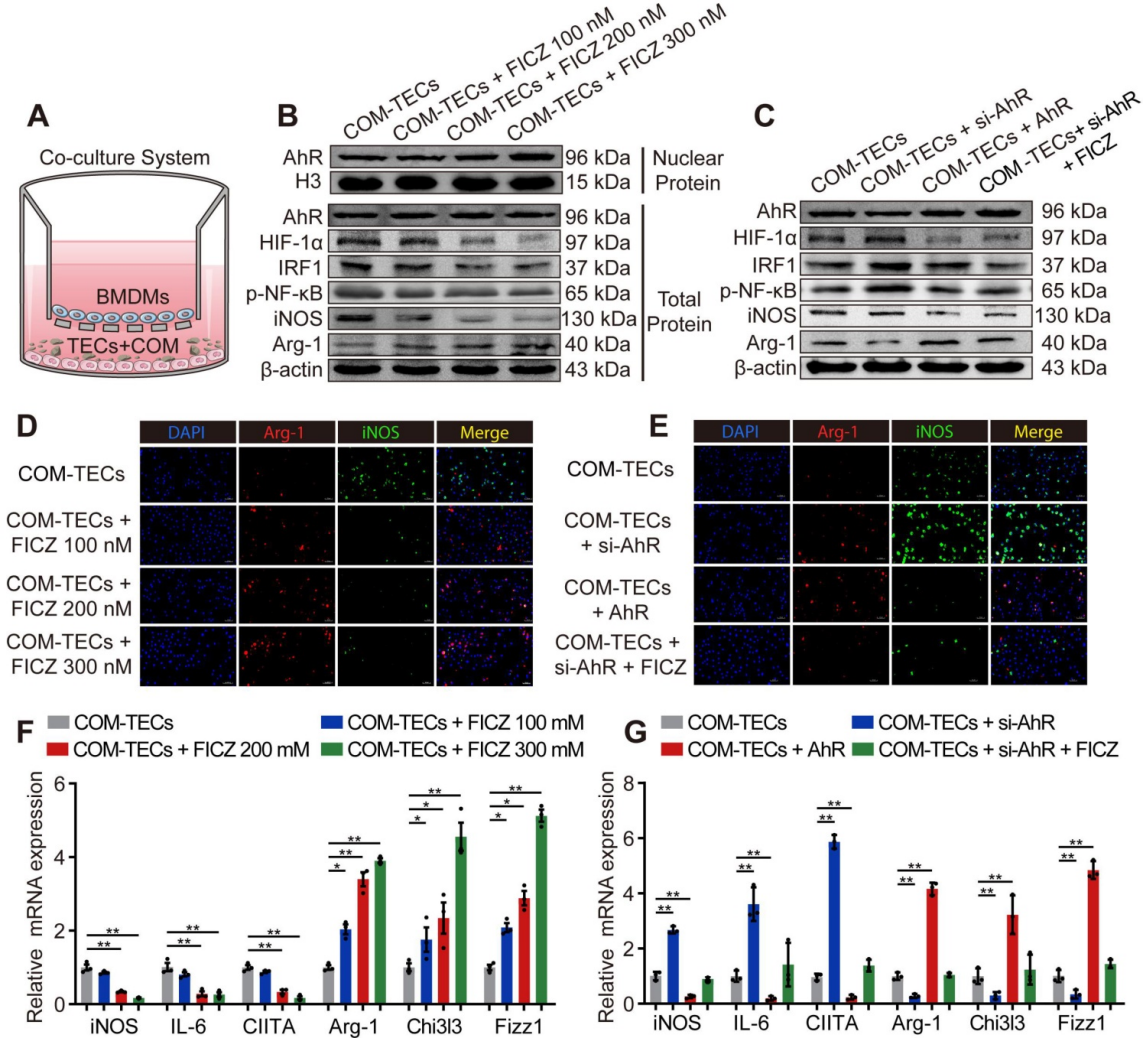
** AhR suppressed IRF1 and HIF-1α to attenuate CaOx crystal-stimulated M1 macrophage polarization *in vitro*.** (A) BMDMs and COM-treated TECs co-culture model. (B, C) Western blotting analysis was used to detect AhR, HIF-1α, IRF1, NF-κB p65, iNOS, and Arg-1 expression after FICZ treatment and the upregulation or downregulation of AhR in BMDMs. β-actin served as a normalization control. (D, E) iNOS (M1 macrophage marker, green) and Arg-1 (M2 macrophage marker, red) distribution in BMDMs were detected by immunofluorescence (200×; scale bar: 20 µm). (F, G) qRT-PCR analysis of iNOS, IL-6, CIITA, Arg-1, Chi3l3 and Fizz1 expression to further determine polarization state of BMDM. The data are shown as the means ± SD of triplicate experiments. *P < 0.05; **P < 0.01, as assessed via one-way ANOVA (F, G).

BMDMs and COM-pretreated TECs (COM-TECs) were co-cultured and treated with FICZ (100 nM, 200 nM, or 300 nM) (Figure 3A). Figure 3B, F and Figure S4A revealed that FICZ promoted the expression of AhR, which in turn suppressed HIF-1α, IRF1, NF-κB p65, and iNOS, IL-6, CIITA (M1 macrophage markers) expression and enhanced Arg-1, Chi3l3, Fizz1 (M2 macrophage marker) levels in BMDMs. BMDMs immunofluorescent staining also showed that FICZ treatment reduced iNOS expression and enhanced Arg-1 expression (Figure 3D, Figure S4B). To confirm that AhR activation within COM-TECs controlled the polarization of macrophages, BMDMs were transfected with an AhR overexpression vector or an AhR siRNA (siAhR). In line with our above findings, AhR and siAhR significantly suppressed and enhanced COM-TECs-mediated M1 macrophage polarization, respectively (Figure 3C, E, G, Figure S4C, D).

### AhR transcriptionally activates miR-142a to inhibit IRF1 and HIF-1α expression

**Figure 4:**
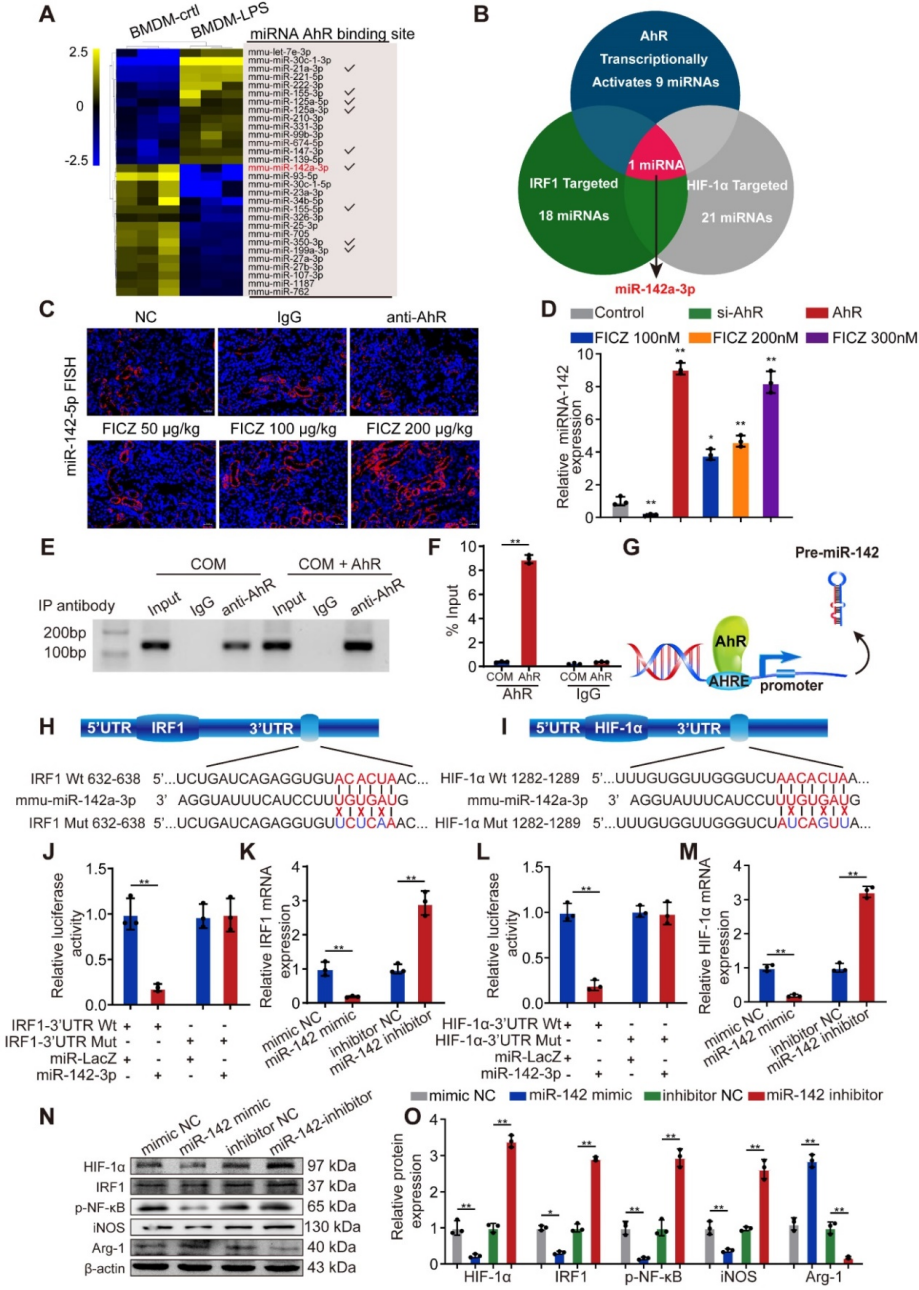
** AhR transcriptionally activates miR-142a to inhibit IRF1 and HIF-1α expression.** (A) The top 30 miRNAs in BMDMs that are regulated by LPS are arranged in a miRNA array heatmap. In addition, miRNAs predicted to be under the transcriptional control of AhR (according to analysis with the JASPAR database) are noted. (B) Venn diagram analyses were performed to identify miRNAs that can both target IRF1 and HIF-1α and that are under the transcriptional control of AhR. (C) Renal expression of mmu-miR-142a-3p in mice (n = 6) with CaOx nephrocalcinosis following treatment with an AhR neutralizing antibody or FICZ treatment was assessed via FISH (200×; scale bar: 20 µm). (D) qRT-PCR was performed to measure mmu-miR-142a-3p expression in BMDMs using U6 RNA as a normalization control. (E, F) ChIP assays and ChIP qPCR analysis showed that AhR bound to the miR-142a promoter in BMDMs treated with the AhR overexpression plasmid. (G) A schematic model showed that AhR directly binds to the miR-142a promoter and activates its transcription. (H, I) WT and mutated miR-142a targeting sequences in the IRF1 and HIF-1α 3'-UTR regions that were used to construct luciferase reporters, with reporters bearing these IRF1 (J) or HIF-1α (L) 3'-UTR sequences co-transfected along with miR-142a mimic (100 nM). IRF1 (K) and HIF-1α (M) mRNA levels were detected via qRT-PCR in BMDMs following miR-142a mimic or inhibitor transfection. Western blotting (N, O) analysis enabled the detection of IRF1 and HIF-1α expression while also assessing the levels of iNOS and Arg-1 to monitor the polarization state of BMDMs following miR-142a mimic or inhibitor transfection. β-actin was employed as a normalization control. The data are shown as the means ± SD of triplicate experiments. *P < 0.05; **P < 0.01, as assessed via Student's t test (D, F) or one-way ANOVA (J-M, O).

MiRNAs can be activated or silenced by specific transcription factors and play crucial roles in posttranscriptional regulation. To further illuminate the mechanism whereby AhR activation suppresses CaOx-mediated cell injury via miRNAs, we reviewed GEO datasets and identified a dataset analysing LPS-induced miRNAs in BMDMs (GSE98810), with the top 30 miRNAs controlled by LPS in these cells being identified through heatmap and clustering analyses. JASPAR database analyses revealed that 9 of these miRNAs had the potential to be activated by AhR by binding to their corresponding promoters (Figure 4A). Next, we found 18 and 21 miRNAs that were predicted to target IRF1 and HIF-1α, respectively, by utilizing the miRanda, miRWalk, TargetScan, and RNA22 databases (Table S2). Of the 9 miRNAs shown in Figure 4A, Venn diagrams revealed that only miR-142a-3p can target HIF-1α and IRF1 while also being controlled at the transcriptional level by AhR (Figure 4B).

To further determine whether AhR activation affects miR-142a expression, we employed a FISH staining approach in murine kidney tissue and assessed BMDMs via qRT-PCR. FICZ promoted the expression of miR-142a, whereas miR-142a expression was inhibited in mice following the administration of an antibody that neutralized AhR and in BMDMs after siAhR treatment (Figure 4C, D). To explore the mechanistic basis for this finding, we used JASPAR to identify putative AHREs within 2 kb of the transcriptional start site of miR-142a, revealing an AHRE consensus-binding site (5′-TTCCACGCAACATGG-3′). Subsequent ChIP analyses demonstrated that AhR binds to AHRE in the miR-142a promoter to promote its transcription (Figure 4E-G).

The 3′-UTRs of IRF1 and HIF-1α were found to harbour predicted miR-142a-3p-binding sites (Figure 4H, I). We therefore generated luciferase reporter plasmids containing IRF1 or HIF-1α with a wild-type or mutant miR-142a binding 3'-UTR site. Luciferase activity in the cells co-transfected with the wt-IRF1 or wt-HIF-1α vector and miR-142a mimic was significantly decreased relative to the control cells (Figure 4J, L). In line with this, qRT-PCR and Western blotting analysis showed that transfection with miR-142a mimic and inhibitor strikingly decreased and increased IRF1 and HIF-1α expression in BMDMs (Figure 4K, M-O). In addition, qRT-PCR revealed that miR-142a mimic inhibited M1 macrophage polarization and improved M2 macrophage polarization, while the miR-142a inhibitor had the opposite effect (Figure S5A).

### AhR activation decrease M1 macrophage polarization to inhibit kidney inflammation and injury through the AhR-miR-142a-IRF1/HIF-1α axis *in vitro*

**Figure 5:**
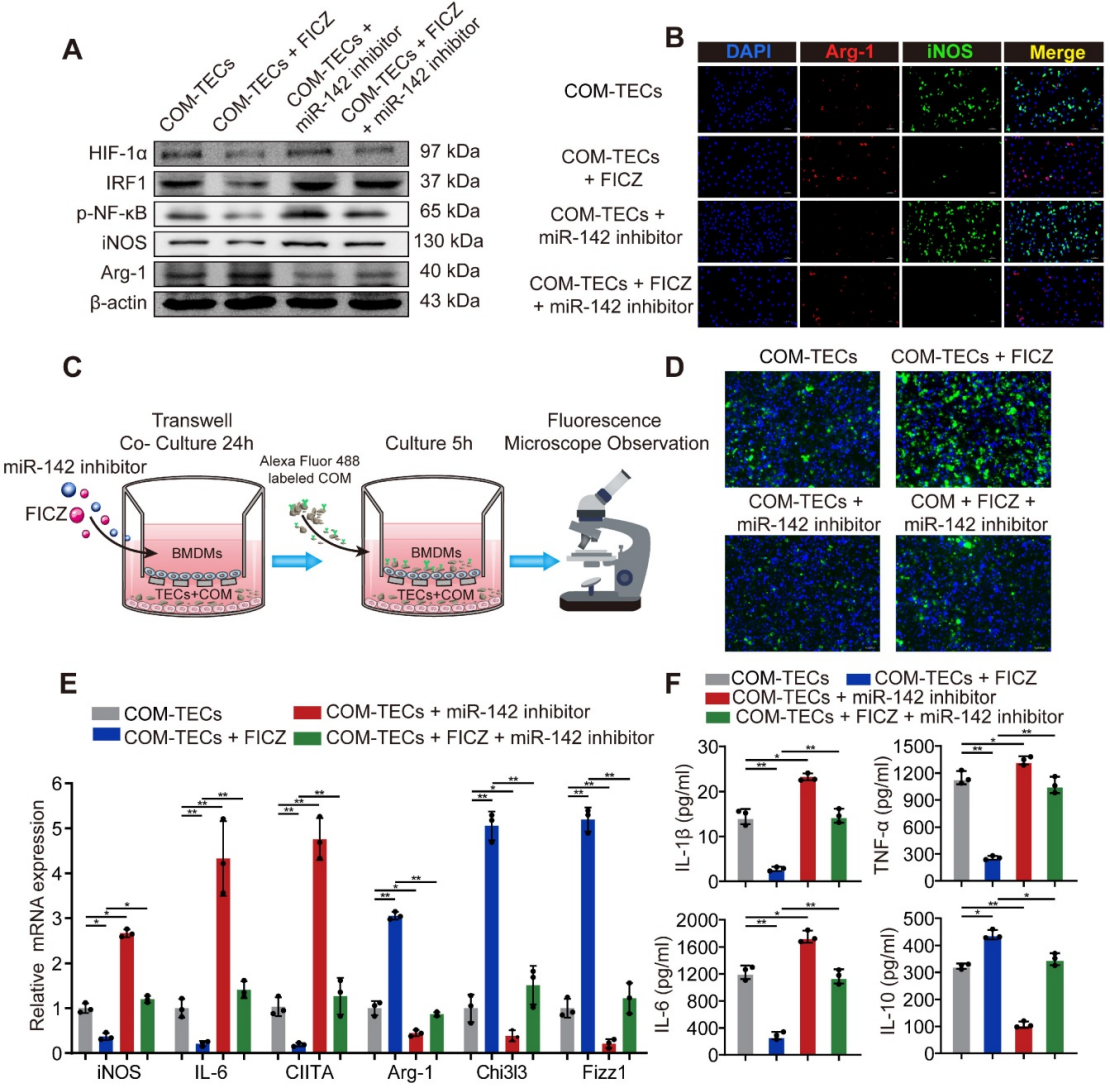
** AhR activation *in vitro* decrease M1 macrophage polarization to inhibit kidney inflammation and injury through the AhR-miR-142a-IRF1/HIF-1α axis *in vitro*.** (A) Western blotting analysis enabled the detection of AhR, HIF-1α, IRF1, NF-κB p65, iNOS, and Arg-1 expression in BMDMs. β-actin was detected as an internal control. (B) iNOS (M1 macrophage marker, green) and Arg-1 (M2 macrophage marker, red) distributions in BMDMs were detected by immunofluorescence (200×; scale bar: 20 µm). (C) Schematic diagram of BMDMs phagocytic capacity testing. (D) Fluorescence microscopy was performed to analyse the phagocytic ability of BMDMs (200×; scale bar: 20 µm). (E) qRT-PCR analysis of iNOS, IL-6, CIITA, Arg-1, Chi3l3 and Fizz1 expression to further determine polarization state of BMDM. (F) ELISA was used to quantify cytokine levels in the co-culture media. The data are shown as the means ± SD of triplicate experiments. *P < 0.05; **P < 0.01, as assessed via one-way ANOVA (E, F).

M2 macrophages are associated with tissue regrowth and with anti-inflammatory activities. To assess the ability of AhR activation to decrease M1 macrophage, polarization and drive M2 macrophage polarization to suppress CaOx crystal-mediated inflammatory damage via the AhR-miR-142a-IRF1/HIF-1α axis *in vitro* was investigated. BMDMs were initially treated with an miR-142a inhibitor and/or FICZ and co-cultured with COM-treated TECs. FICZ treatment suppressed HIF-1α, IRF1, M1 macrophage markers iNOS and promoted the M2 macrophage marker Arg-1, whereas the miR-142a inhibitor had the opposite effects (Figure 5A, Figure S6A). Immunofluorescent staining and qRT-PCR analysis similarly showed that miR-142a inhibitor partially reversed AhR activation-driven M2 macrophage polarization (Figure 5B, E, Figure S6B). We also assessed BMDMs phagocytosis ability via fluorescence microscopy. As we expected, FICZ treatment made BMDMs increase rate of COM crystal phagocytosis, whereas miR-142a inhibitor caused the opposite effect (Figure 5C, D, Figure S6C). ELISA was thus performed to detect the levels of pro-inflammatory and anti-inflammatory cytokines in the media, and we found that IL-1β, TNF-α, and IL-6 levels were strikingly suppressed, while IL-10 production was enhanced in the FICZ-treated group. In addition, BMDM treatment with FICZ and a miR-142a inhibitor revealed that the miR-142a inhibitor partially reversed the protective effects of AhR activation (Figure 5F).

### AhR activation suppressed CaOx crystal deposition and CaOx nephrocalcinosis-mediated kidney inflammation and injury through the AhR-miR-142a-IRF1/HIF-1α axis *in vivo*

**Figure 6:**
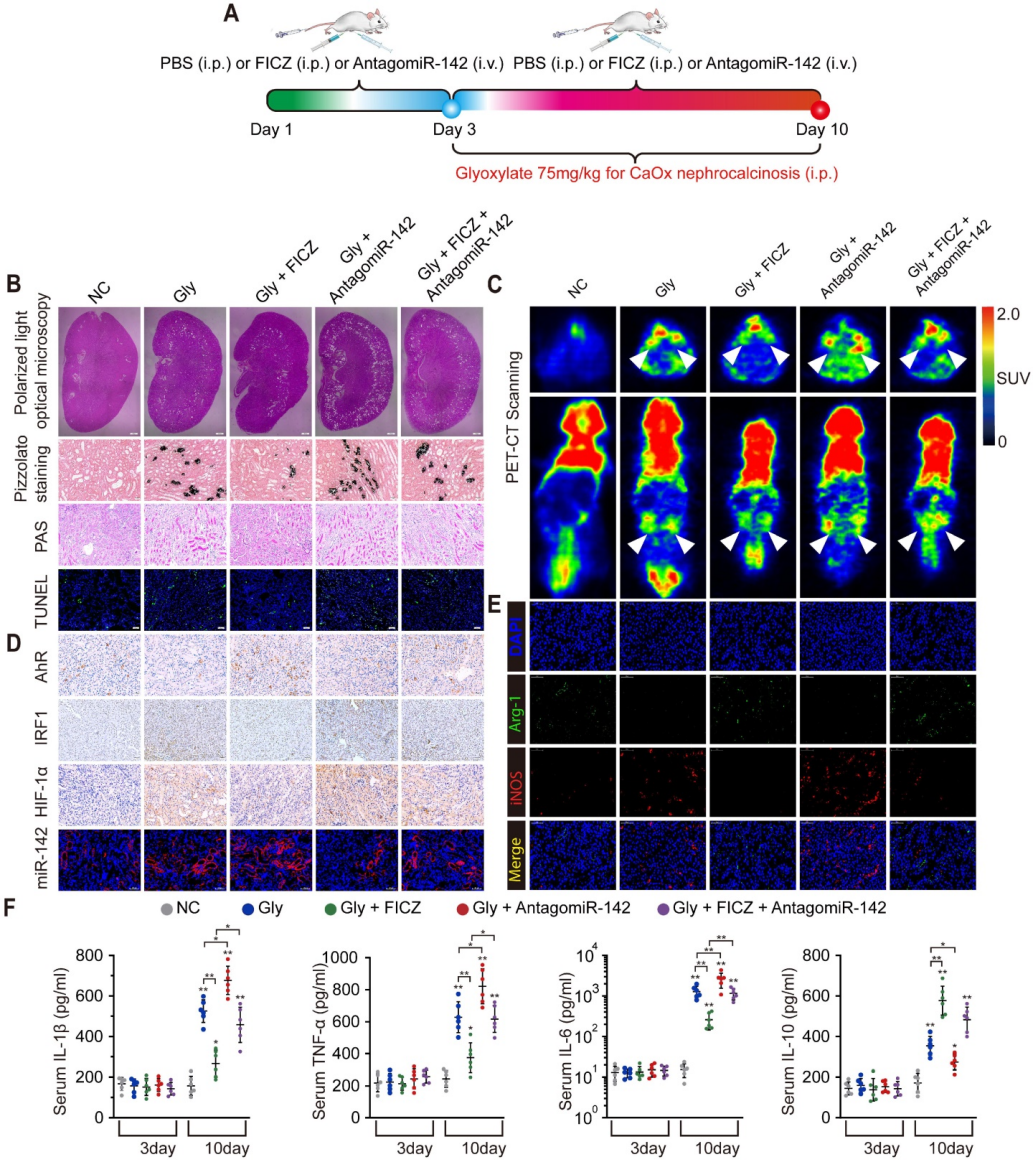
** AhR activation suppressed the deposition of CaOx crystal and CaOx nephrocalcinosis-mediated kidney inflammation and injury through the AhR-miR-142a-IRF1/HIF-1α axis *in vivo*.** (A) Experimental overview. (B) The deposition of renal CaOx crystal in FICZ- and/or antagomiR-142a-treated mice was assessed via polarized light optical microscopy (20×; scale bar: 500 µm). Pizzolato staining was employed as a means of detecting these CaOx crystal in corticomedullary tissue, while PAS was utilized to evaluate injury to TECs (200×; scale bar: 20 µm), and TUNEL staining was employed to assess renal TECs death (200×; scale bar: 50 µm). (C) PET-CT scanning was employed as a means of assessing renal inflammation state in CaOx nephrocalcinosis mice. (D) IHC was used to analyse AhR, IRF1, and HIF-1α expression, and FISH was used to detect miR-142a expression in renal tissue (200×; scale bar: 20 µm). (E) iNOS (M1 macrophage marker, red) and Arg-1 (M2 macrophage marker, green) distributions in renal tissues were detected by immunofluorescence (200×; scale bar: 50 µm). (F) On days 3 and 10, the serum pro-inflammatory IL-1β, TNF-α, and IL-6 levels and the anti-inflammatory IL-10 levels were measured by ELISA. n = 6 per group. *P < 0.05; **P < 0.01, as assessed via one-way ANOVA (F).

Widespread macrophage infiltration, as well as TECs necrosis, was associated with the deposition of CaOx crystal in the kidney. To evaluate how AhR activation suppresses CaOx crystal deposition and CaOx nephrocalcinosis-mediated renal damage *in vivo*, mice were treated for 3 days with FICZ and/or antagomiR-142a, after which a CaOx nephrocalcinosis model was generated using Gly for 7 days (Figure 6A). Pizzolato staining and polarized light optical microscopy showed that FICZ treatment strikingly reduced CaOx crystal in cortex medullary junction but antagomiR-142a caused the opposite effect. TUNEL and PAS staining further revealed that antagomiR-142a suppressed the impact of FICZ treatment on the prevention of TECs injury and necrosis (Figure 6B, and Figure S7A-D). To examine local renal inflammatory responses, we next analysed ^18^F-FDG uptake. Micro-PET-CT imaging revealed that FICZ treatment markedly decreased ^18^F-FDG uptake, while antagomiR-142a bolstered these parameters (Figure 6C). Robust AhR IHC staining and miR-142a (FISH) together with weak renal intestinal HIF-1α and IRF1 staining were detected in FICZ-treated mice (Figure 6D, Figure S7E-H). To further exclude the possibility that FICZ interferes with the CaOx crystal formation, we established CaOx nephrocalcinosis mouse model and then treated them with FICZ. Consist with previous results; FICZ treatment strikingly reduced renal CaOx crystal and renal injury while AhR increased (Figure S8).

FICZ treatment markedly suppressed glyoxylate-induced renal inflammatory damage and necrotic death, potentially owing to its impact on macrophage polarization. Immunofluorescence assays showed that exacerbated renal interstitial inflammation and increased M1 macrophage (iNOS) infiltration were observed in antagomiR-142a-treated mice with nephrocalcinosis compared with FICZ-treated mice with nephrocalcinosis (Figure 6E). Furthermore, the levels of IL-1β, IL-6, TNF-α, and IL-10, serum creatinine and BUN were determined to evaluate inflammation state and renal function in our mouse model after different treatments. With FICZ or antagomiR-142a treatment, increased inflammatory cytokine levels and a decrease in renal function were observed in the glyoxylate injection group. However, FICZ treatment preserved kidney function and enhanced the secretion of IL-10, whereas the opposite phenotype was associated with antagomiR-142a treatment (Figure 6F and Table S3). Under normal circumstances, the level of serum IL-10 begins to decrease after 24-28 hours and gradually becomes normal in mouse acute kidney injury model 26. It is possible that only when the risk factors are eliminated will IL-10 levels start to decrease significantly. In our study, the IL-10 level remained at a high level in the 10th day, which may be because the mice were still injected with glyoxylate which caused acute kidney injury at this time.

Together, these findings indicated that AhR activation could diminish M1 macrophage polarization and promote M2 macrophage polarization to suppress CaOx nephrocalcinosis-mediated renal damage and CaOx crystal deposition through the AhR-miR-142a-IRF1/HIF-1α pathway.

## Discussion

CaOx-mediated kidney injury and crystal deposition are primary drivers of nephrocalcinosis 27, 28. Kidney CaOx crystal induce localized injury and inflammation through direct contact with the tubular epithelium 28, 29. In turn, the renal inflammatory response is a key regulator of the development of nephrolithiasis 27.

Our previous data showed that the exposure of TECs to CaOx crystal can promote excessive reactive oxygen species production and pro-inflammatory cytokine secretion, leading to kidney injury 5, 12, 13. During the development of nephrolithiasis, renal epithelial cells secrete monocyte chemoattractant protein-1 (MCP-1) to recruit macrophages to the renal interstitium around the interstitial crystal 30, 31. In addition, crystal deposition can increase inflammatory gene expression, in turn driving the migration and phagocytic activity of monocytes and macrophages 32. Recent studies have shown that macrophages regulate crystal deposition in the kidney interstitium. Pro-inflammatory M1 macrophages accelerate renal damage and crystal deposition, while anti-inflammatory M2 macrophages suppress crystal deposition via the phagocytic uptake of CaOx crystal and the inhibition of oxidation-induced renal damage 9, 11, 32, 33. While many studies have attempted to reveal the important regulators in inflammation-associated tissue damage and the regulation of macrophage polarization 17, 34-36, the mechanism underlying crystal-related kidney injury and inflammation remains obscure.

Macrophages that are activated by endogenous and exogenous pathogens are differentiated and polarized into distinct phenotypes through the activation of Toll-like receptors (TLRs), nucleotide-binding oligomerization domain (NOD)-like receptors (NLRs) and other receptors 37, 38. The biological significance of AhR modulate macrophage polarization via these downstream inflammatory signals was documented in several contexts. Toll-like receptor 4 (TLR4) inhibition decreases M1 and enhances M2 macrophage polarization 13, 37, 39. AhR was demonstrated to modulate iNOS and TLR4 expression by competing with hypoxia inducible factor 1-alpha (HIF-1α) for binding to aryl hydrocarbon nuclear translocator (ARNT) 40. AhR was also reported to play important roles in Toll-like receptor 9 (TLR9), TLR4 and interferon-regulatory factor 4 (IRF4) signalling to suppress M1 macrophage polarization 41-44. Likewise, blockade of the nucleotide-binding domain, leucine-rich-containing family, pyrin domain-containing-3 (NLRP3) attenuates M1 macrophage polarization 45, 46. Zhao et al. reported that AhR could bind to the xenobiotic response element (XRE) region located in the promoter of NLRP3 and inhibit NLRP3 transcription and subsequent inflammasome activation 47. Batist et al. found that nuclear factor erythroid 2-related factor 2 (Nrf2) gene transcription is directly modulated by AhR activation 48. Our previous study demonstrated that Nrf2 activation suppressed M1 macrophage polarization and increased M2 macrophage polarization by downregulating TLR4 and interferon-regulatory factor 1 (IRF1), which provides clues for the crosstalk between AhR, Nrf2 and IRF1 13. However, Sogawa et al. found that Nrf2-dependent NLRP3 inflammasome activation was essential for maintaining M1 macrophage polarization 49. Therefore, the roles of Nrf2 in macrophage polarization remain controversial. In addition, it has been reported that 2,3,7,8-Tetrachlorodibenzo-p-dioxin (TCDD), an AhR-related inducer of the cytochrome P450 1A1 (CYP1A1), enhances the DNA binding activity of nuclear factor-kappaB (NF-κB) and activator protein-1 (AP-1) in macrophages during inflammation or sepsis 50, 51. In summary, the precise mechanisms by which AhR regulates macrophage polarization, especially in CaOx nephrocalcinosis, remain ambiguous and require further investigation. Here, we mainly focused on the regulation of HIF-1α and IRF1 (two crucial M1 macrophage polarization factors) by AhR through a post-transcriptional mechanism.

A network of signalling molecules, transcription factors, epigenetic mechanisms, and post-transcriptional regulators underlies macrophage polarization 52. IRF1 and HIF-1α have been identified as powerful regulators of macrophage activation and polarization 53-56. These two genes have been demonstrated to play pathogenic roles in the context of kidney injury and other diseases. IRF1 is a powerful regulator of M1 macrophage polarization 12, 13. The activation of IRF1 is crucial for the increase in iNOS expression 57, 58. Additionally, HIF-1α is differentially expressed in M1- and M2-polarized macrophages 59 and is a transcriptional activator of iNOS (M1 gene) expression 60, 61. Arginase1 (Arg-1), regarded as one of the most specific markers of M2 macrophages, is highly expressed in M2 macrophages and competes with iNOS for a common substrate, L-arginine 62, 63. AhR may indirectly increase Arg-1 expression by inhibiting iNOS expression via the miR-142a-IRF1/HIF-1α pathway.

As our previous data showed, Nrf2 exerted an inhibitory effect of IRF1 to decrease M1 macrophage polarization via miR-93 13. In addition, AhR was reported to directly promote Nrf2 transcription 48. Collectively, these studies indicate that IRF1 is an important regulator of M1 macrophage polarization controlled by different upstream transcription factors. IRF1 might potentially serve as a therapeutic target for the regulation of macrophage polarization and CaOx-induced nephrocalcinosis. Therefore, our future studies on exploring the upstream signalling pathway of IRF1 and exploiting inhibitors of IRF1 are of great importance. It has been reported that HIF-1α can induce glomerular injury and chronic kidney disease by activating NF-κB 64. In addition, abnormal activity of HIF-1α in the kidney epithelium accelerates interstitial fibrosis 65. Consistent with our current findings, AhR activation downregulates IRF1 and HIF-1α via miR-142a, which decreases M1 macrophage polarization, increases M2 macrophage polarization, and reduces pro-inflammatory cytokine levels to ultimately protect against CaOx nephrocalcinosis-mediated kidney injury.

MicroRNAs (miRNAs) are endogenous small RNAs that are approximately 22 nt in length. They play crucial negative regulatory roles in inducing the degradation or translational repression of protein-coding mRNAs 66, 67. Increasing evidence has shown that miRNAs play important roles in regulating macrophage-mediated inflammatory responses, including macrophage activation and polarization, tissue infiltration, resolution of inflammation, and kidney injury 68-70. Researchers have revealed that miR-142 regulates profibrogenic macrophage gene expression in chronic inflammation 71. Talebi et al. demonstrated that miR-142 regulates autoimmune neuroinflammation and T cell differentiation in an animal model of multiple sclerosis 72. MiR-142 has also been reported to suppress macrophage activation and lung inflammation via inhibition of nlrp3 inflammasome activation 73. Similarly, we found that miR-142a inhibited CaOx nephrocalcinosis-mediated kidney injury by targeting IRF1 and HIF-1α to suppress M1 macrophage polarization and promote M2 macrophage polarization.

In summary, we demonstrated that AhR activation in macrophages plays a protective role in CaOx nephrocalcinosis-mediated kidney injury and crystal deposition. We also revealed the mechanism whereby the AhR-miR-142a interaction regulates the IRF1/HIF-1α pathway. The present study offers new mechanistic insight into the regulation of macrophages during CaOx nephrocalcinosis and may accelerate the development of new therapeutic interventions to target renal CaOx crystal deposition and reduce nephrocalcinosis-mediated kidney inflammation and injury.

## Supplementary Material

Supplementary figures and tables.

## Abbreviations

CaOx: calcium oxalate
AhR: aryl hydrocarbon receptor
Gly: glyoxylate
FICZ: 6-formylindolo(3, 2-b)carbazole
PET-CT: positron emission tomography computed tomography
18F-FDG: 18F-fluorodeoxyglucose
SUVs: standardized uptake-values
ROI: regions of interest
BUN: blood urea nitrogen
PAS: Periodic acid-Schiff
TUNEL: terminal deoxynucleotidyl transferase dUTP nick end labeling
IHC: immunohistochemistry
FISH: fluorescent *in situ* hybridization
TECs: renal tubular epithelial cells
BMDMs: bone marrow-derived macrophages
M-CSF: macrophage colony-stimulating factor
COM: calcium oxalate monohydrate
qRT-PCR: real-time quantitative reverse transcriptase-polymerase chain reaction
LDH: lactate dehydrogenase
ELISA: enzyme linked immunosorbent assay
ChIP: Chromatin Immunoprecipitation
3'-UTR: 3'-untranslated region
RNA-seq: RNA sequencing
GO: gene ontology
MCP-1: monocyte chemoattractant protein-1
TLRs: Toll-like receptors
NOD: nucleotide-binding oligomerization domain
NLRs: nucleotide-binding oligomerization domain (NOD)-like receptors
TLR4: Toll-like receptor 4
HIF‐1α: hypoxia inducible factor 1-alpha
ARNT: aryl hydrocarbon nuclear translocator
TLR9: Toll-like receptor 9
IRF4: interferon-regulatory factor 4
NLRP3: nucleotide-binding domain, leucine-rich-containing family, pyrin domain-containing-3
XRE: xenobiotic response element
Nrf2: nuclear factor erythroid 2-related factor 2
IRF1: interferon-regulatory factor 1
TCDD: 2,3,7,8-Tetrachlorodibenzo-p-dioxin
CYP1A1: cytochrome P450 1A1
NF-κB: nuclear factor-kappaB
AP-1: activator protein-1
Arg-1: Arginase1
miRNAs: microRNAs
