# Supplementary material for: AhR activation attenuates calcium oxalate nephrocalcinosis by diminishing M1 macrophage polarization and promoting M2 macrophage polarization
Source: Theranostics. 2020 Oct 25;10(26):12011–25. doi: 10.7150/thno.51144 (PMC7667681; doi:10.7150/thno.51144)
Supplement: Supplementary file 1 — Supplementary figures and tables. [file thnov10p12011s1.pdf]

Supplemental Material

Table S1. List of primer sequences used for the qRT-PCR analysis

Name	(5'-3')	SEQUENCE (5'-3')
AhR	Forward	CAAATCCTTCTAAGCGACACAG
	Reverse	TGACGCTGAGCCTAAGAACA
HIF-1α	Forward	GATGACGGCGACATGGTTTAC
	Reverse	CTCACTGGGCCATTTCTGTGT
IRF1	Forward	TTGGCATCATGGTGGCTGT
	Reverse	AAGGAGGATGGTCCCCTGTTT
iNOS	Forward	CACCTTGGAGTTCACCCAGT
	Reverse	ACCACTCGTACTTGGGATGC
IL-6	Forward	TAGTCCTTCCTACCCCAATTTCC
	Reverse	TTGGTCCTTAGCCACTCCTTC
CIITA	Forward	TGCGTGTGATGGATGTCCAG
	Reverse	CCAAAGGGGATAGTGGGTGTC
Arg-1	Forward	TGGCTTGCGAGACGTAGAC
	Reverse	GCTCAGGTGAATCGGCCTTTT
Chi3l3	Forward	CAGGTCTGGCAATTCTTCTGAA
	Reverse	GTCTTGCTCATGTGTGTAAGTGA
Fizz1	Forward	CCAATCCAGCTAACTATCCCTCC
	Reverse	ACCCAGTAGCAGTCATCCCA
β-actin	Forward	CTGAGAGGGAAATCGTGCGT
	Reverse	CCACAGGATTCCATACCCAAGA

Table S2. Selected miRNAs targeted IRF1 or HIF-1α positively predicted by the four database.

Gene	RefseqID	miRNA	MIMATid	miRWalk	miRanda	RNA22	Targetscan	SUM
IRF1	NC_000077.6	mmu-miR-140-3p.1	MIMAT0000152	1	1	1	1	4
IRF1	NC_000077.6	mmu-miR-130a-3p	MIMAT0016983	1	1	1	1	4
IRF1	NC_000077.6	mmu-miR-130b-3p	MIMAT0004583	1	1	1	1	4
IRF1	NC_000077.6	mmu-miR-301a-3p	MIMAT0017008	1	1	1	1	4
IRF1	NC_000077.6	mmu-miR-301b-3p	MIMAT0017232	1	1	1	1	4
IRF1	NC_000077.6	mmu-miR-23a-3p	MIMAT0017019	1	1	1	1	4
IRF1	NC_000077.6	mmu-miR-23b-3p	MIMAT0016980	1	1	1	1	4
IRF1	NC_000077.6	mmu-miR-9-5p	MIMAT0000142	1	1	1	1	4
IRF1	NC_000077.6	mmu-miR-383-5p	MIMAT0000748	1	1	1	1	4
IRF1	NC_000077.6	mmu-miR-17-5p	MIMAT0000649	1	1	1	1	4
IRF1	NC_000077.6	mmu-miR-20a-5p	MIMAT0000529	1	1	1	1	4
IRF1	NC_000077.6	mmu-miR-20b-5p	MIMAT0003187	1	1	1	1	4
IRF1	NC_000077.6	mmu-miR-93-5p	MIMAT0000540	1	1	1	1	4
IRF1	NC_000077.6	mmu-miR-106a-5p	MIMAT0000385	1	1	1	1	4
IRF1	NC_000077.6	mmu-miR-106b-5p	MIMAT0000386	1	1	1	1	4
IRF1	NC_000077.6	mmu-miR-203-3p	MIMAT0000236	1	1	1	1	4
IRF1	NC_000077.6	mmu-miR-124-3p	MIMAT0000134	1	1	1	1	4
IRF1	NC_000077.6	mmu-miR-142a-3p	MIMAT0000155	1	1	1	1	4
HIF1α	NC_000077.6	mmu-miR-99a-5p	MIMAT0000229	1	1	1	1	4
HIF1α	NC_000077.6	mmu-miR-135b-5p	MIMAT0000612	1	1	1	1	4
HIF1α	NC_000077.6	mmu-miR-142a-3p	MIMAT0000155	1	1	1	1	4
HIF1α	NC_000077.6	mmu-miR-126a-3p	MIMAT0000138	1	1	1	1	4
HIF1α	NC_000077.6	mmu-miR-150-5p	MIMAT0000160	1	1	1	1	4
HIF1α	NC_000077.6	mmu-miR-18a-5p	MIMAT0000528	1	1	1	1	4
HIF1α	NC_000077.6	mmu-miR-138-5p	MIMAT0000150	1	1	1	1	4
HIF1α	NC_000077.6	mmu-miR-20a-5p	MIMAT0000529	1	1	1	1	4
HIF1α	NC_000077.6	mmu-miR-17-5p	MIMAT0000649	1	1	1	1	4
HIF1α	NC_000077.6	mmu-miR-93-5p	MIMAT0000540	1	1	1	1	4
HIF1α	NC_000077.6	mmu-miR-106a-5p	MIMAT0000385	1	1	1	1	4
HIF1α	NC_000077.6	mmu-miR-126a-3p	MIMAT0000138	1	1	1	1	4
HIF1α	NC_000077.6	mmu-miR-150-5p	MIMAT0000160	1	1	1	1	4
HIF1α	NC_000077.6	mmu-miR-19a-3p	MIMAT0000651	1	1	1	1	4
HIF1α	NC_000077.6	mmu-miR-155-5p	MIMAT0000165	1	1	1	1	4
HIF1α	NC_000077.6	mmu-miR-25-3p	MIMAT0000652	1	1	1	1	4
HIF1α	NC_000077.6	mmu-miR-32-5p	MIMAT0000654	1	1	1	1	4
HIF1α	NC_000077.6	mmu-miR-92a-3p	MIMAT0000539	1	1	1	1	4
HIF1α	NC_000077.6	mmu-miR-367-3p	MIMAT0003181	1	1	1	1	4
HIF1α	NC_000077.6	mmu-miR-203-3p	MIMAT0000236	1	1	1	1	4
HIF1α	NC_000077.6	mmu-miR-140-3p	MIMAT0000152	1	1	1	1	4

miRWalk:http://zmf.umm.uni-heidelberg.de/apps/zmf/mirwalk2/generetsys-self.html

miRanda: http://www.microrna.org/microrna/getGeneForm.do

RNA22: https://cm.jefferson.edu/rna22/Interactive/

Targetscan: http://www.targetscan.org/vert_71/

Table S3. Serum BUN and Creatinine levels in mouse model (n = 6).

Parameter	Time	NC	Glyoxylate	Glyoxylate+ FICZ	Glyoxylate+AntagomiR-142	Glyoxylate+ FICZ+AntagomiR-142
Serum creatinine (mg/dl)	3rd day	0.165 ± 0.018	0.158 ± 0.020	0.170 ± 0.033	0.158 ± 0.022	0.169 ± 0.028
	10th day	0.180 ± 0.023	0.558 ± 0.038 [a]	0.236 ± 0.035 [b]	0.688 ± 0.028 [b]	0.483 ± 0.036 [c]
Serum BUN (mg/dl)	3rd day	24.19 ± 1.15	25.28 ± 1.28	23.99 ± 0.89	25.36 ± 1.23	24.66 ± 1.56
	10th day	25.25 ± 1.34	65.25 ± 3.98 [a]	38.12 ± 2.28 [b]	76.18 ± 5.21 [b]	48.26 ± 3.98 [c]

Abbreviations: BUN, blood urea nitrogen.

[a] p <0.05 compare with the normal control group

[b] p <0.05 compare with the glyoxylate group

[c] p <0.05 compare with the glyoxylate + FICZ group

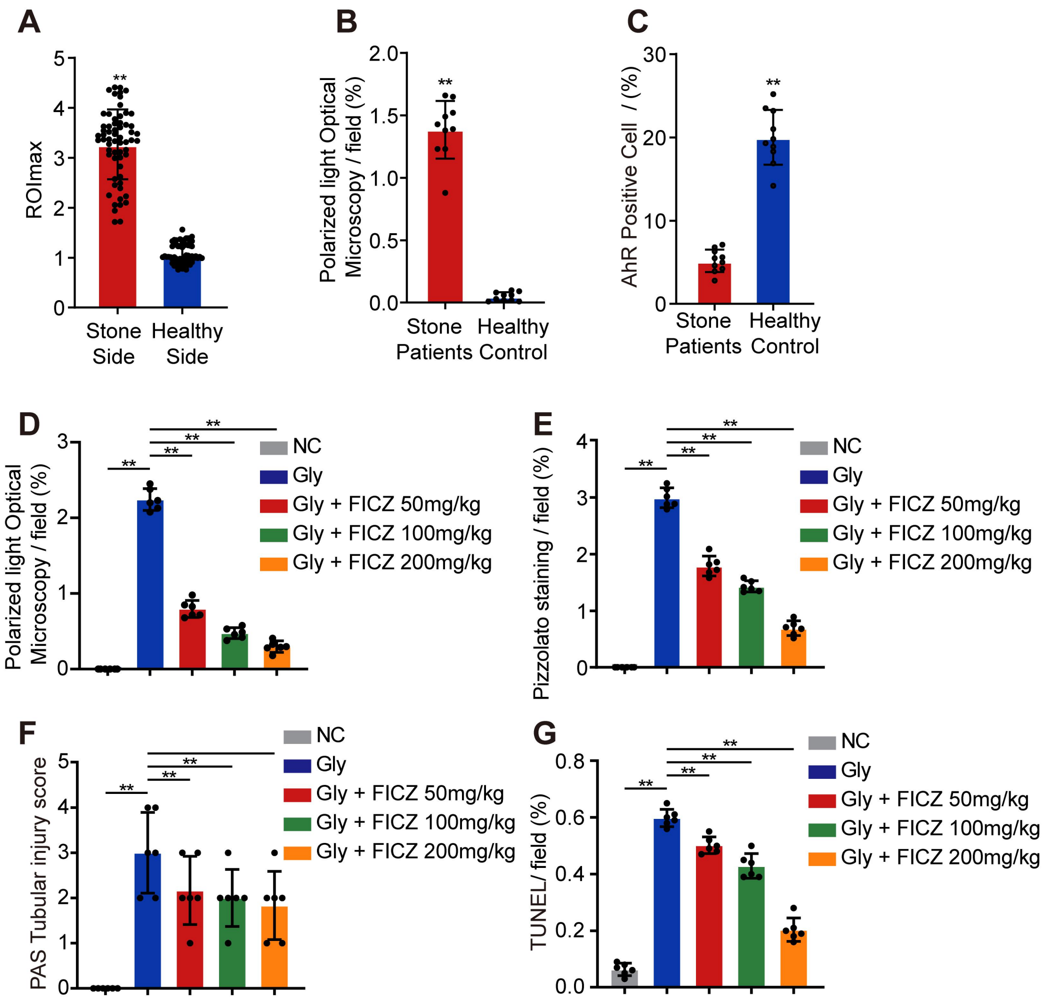

Figure S1. Quantification of ^{18}F-FDG uptake, AhR expression, CaOx crystal deposition, PAS staining, and TUNEL staining. (A) ROI_{max} value of ^{18}F-FDG PET-CT scan in stone patient (n = 62). (B, C) The ratio of areas with renal crystal deposition and IHC positive cells of AhR in Randall's Plague (n = 10). (D) The ratio of areas with renal crystal deposition detected by polarized light optical microscopy. (E) The ratio of areas with kidney corticomedullary junction area crystal deposition detected by Pizzolato staining. (F) The tubular injury score displayed in PAS staining. (G) The average number of TUNEL-positive cells per high power field (200×). (D-G, n = 6). The data are shown as mean ± SD. *P < 0.05; **P < 0.01, as determined by Student's t test (A-C) or one-way ANOVA (D-G).

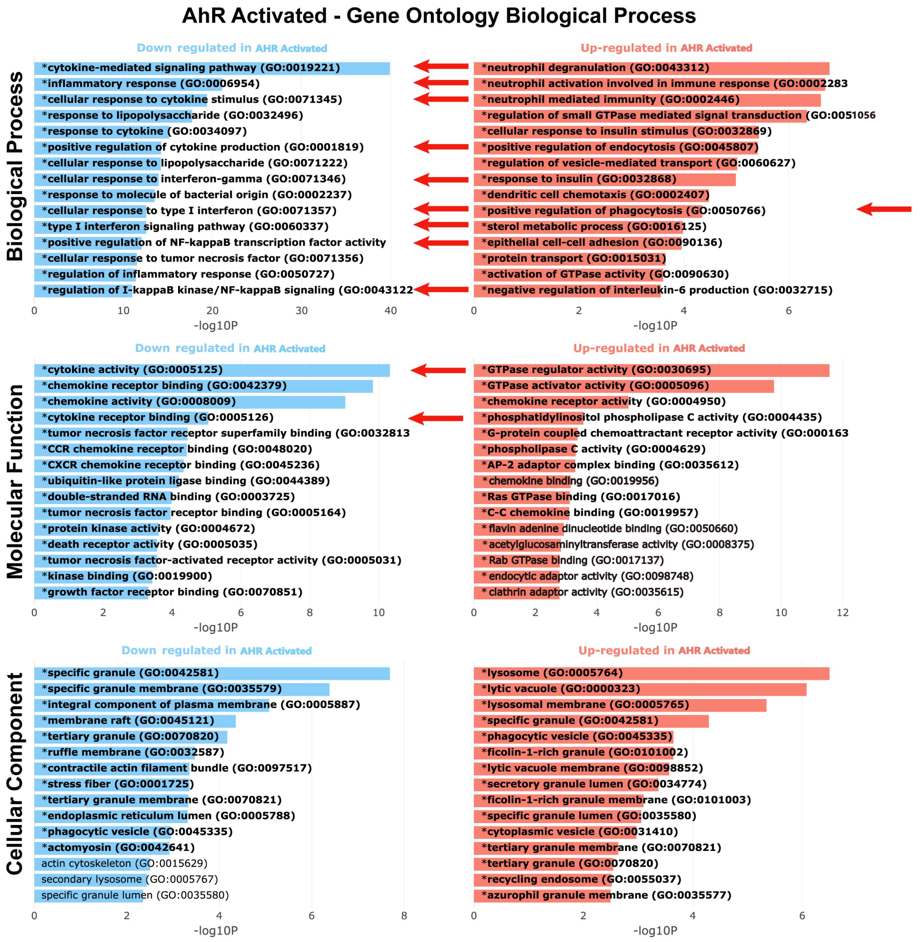

Figure S2. Gene ontology enrichment analysis in AhR activated BMDM. Gene ontology analysis shows that inflammatory response and macrophage activation play a vital role in AhR activated BMDM. cytokine mediated pathway, inflammatory response, type I interferon pathway, macrophage phagocytosis pathway, NF-κB pathway which containing IRF1, HIF-1α, NF-κB, IL-1, IL-6, TNF, Arg-1 which consist with the result in Figure 2.

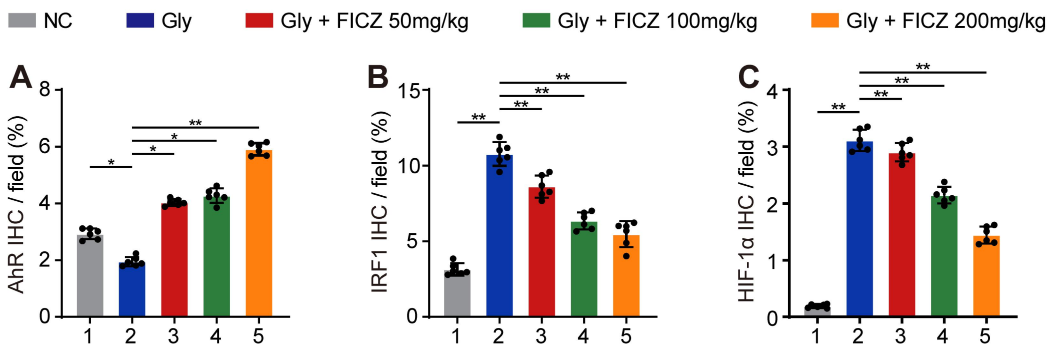

Figure S3. Quantification of IHC in FICZ treated CaOx nephrocalcinosis mouse model. (A-C) The ratio of areas with IHC positive expression of kidney AhR, IRF1, and HIF-1α in CaOx nephrocalcinosis mouse model treated with FICZ at different concentration. n = 6 per group. The data are shown as mean ± SD. *P < 0.05; **P < 0.01, as determined by one-way ANOVA (A-C).

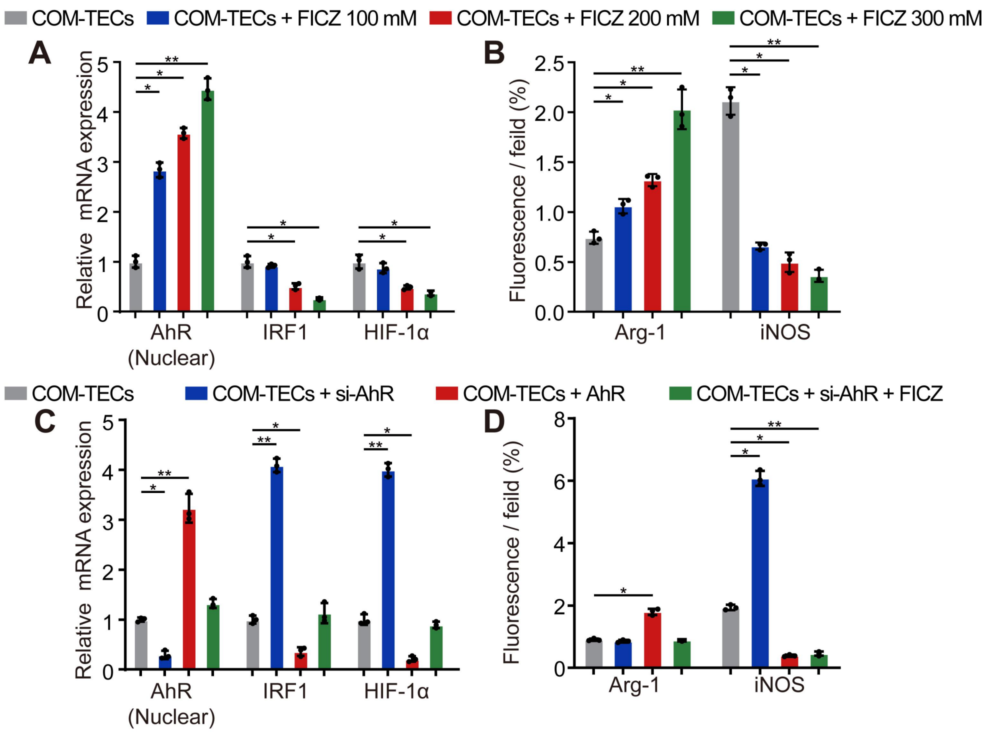

Figure S4. qRT-PCR analysis of AhR, IRF1, and HIF-1α expression, and quantitative immunofluorescence analysis the polarization of BMDMs. (A) qRT-PCR analysis of AhR, IRF1, HIF-1α expression in BMDMs treated with COM crystals treated in the absence or presence of FICZ at different concentration. β-actin was used as an internal control. (B) Immunofluorescence analysis the BMDMs polarization with anti-iNOS and Arg-1. (C) qRT-PCR analysis of AhR, IRF1, HIF-1α, iNOS, and Arg-1 expression in BMDMs treated with COM crystals on the condition of AhR down-regulation and over-expression with FICZ. β-actin was used as an internal control. (D) Immunofluorescence analysis the BMDMs polarization with anti-iNOS and Arg-1. The data are shown as mean ± SD of triplicate experiments. *P < 0.05; **P < 0.01, as determined by one-way ANOVA (A-D).

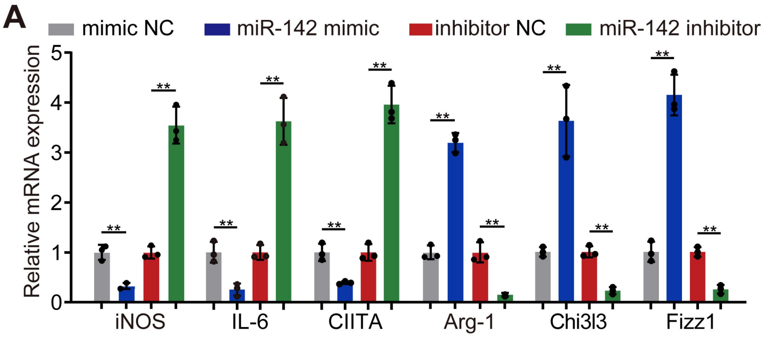

Figure S5. qRT-PCR analysis of the polarization of BMDMs. (A) qRT-PCR analysis of iNOS, IL-6, CIITA, Arg-1, Chi3l3 and Fizz1 expression in BMDMs treated with miR-142 mimics or inhibitor. β-actin was used as an internal control. The data are shown as mean ± SD of triplicate experiments. *P < 0.05; **P < 0.01, as determined by one-way ANOVA .

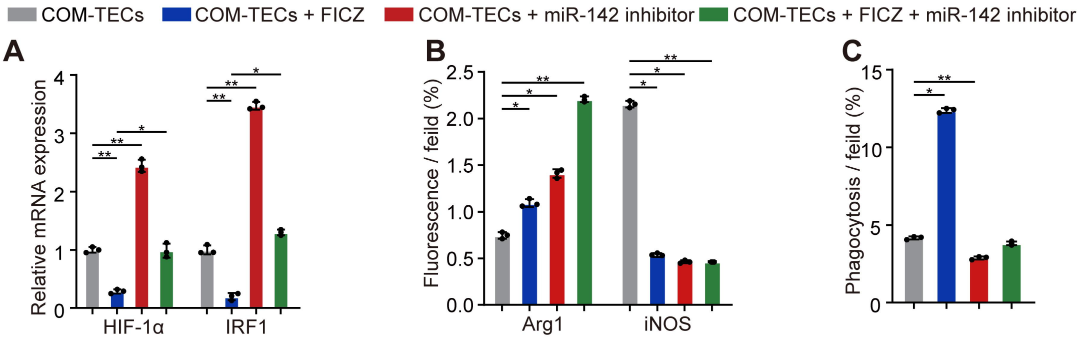

Figure S6. qRT-PCR analysis of HIF-1α and IRF1, quantitative immunofluorescence analysis, and BMDM phagocytosis ratio. (A) qRT-PCR analysis of HIF-1α, IRF1 expression in BMDMs treated with FICZ and/or an miR-142a inhibitor. (B) Immunofluorescence analysis the BMDMs polarization with anti-iNOS and anti-Arg-1. (C) Fluorescence microscopy measure the phagocytic ability of BMDMs. The data are shown as mean ± SD of triplicate experiments. $^{*}P < 0.05$; $^{**}P < 0.01$, as determined by one-way ANOVA (A-C).

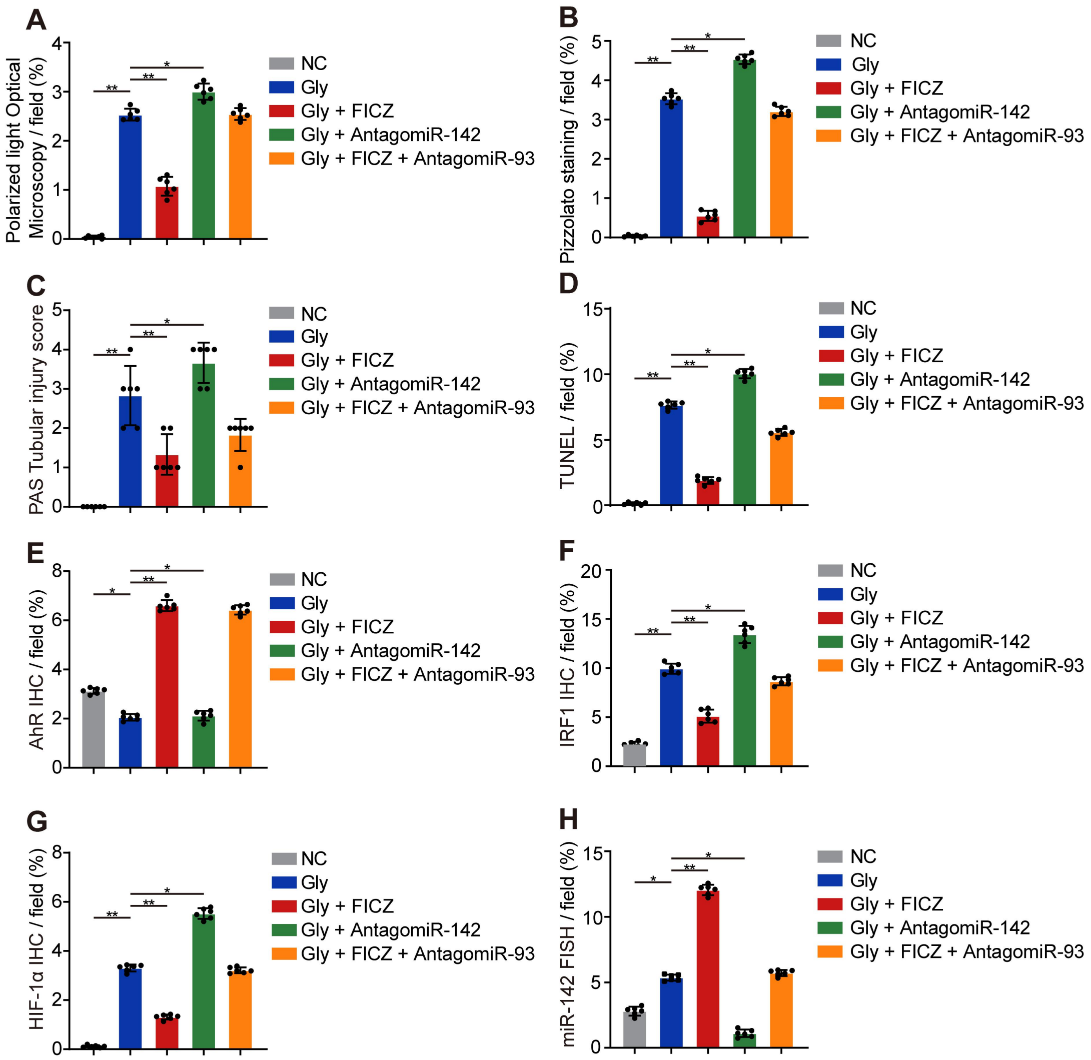

Figure S7. Quantification of CaOx crystal deposition, PAS staining, AhR, IRF1, HIF-1α IHC staining, and miR-142 FISH. (A) The ratio of areas with renal crystal deposition detected by polarized light optical microscopy. (B) The ratio of areas with kidney corticomedullary junction area crystal deposition. (C) The tubular injury score displayed in PAS staining. (D) The average number of TUNEL-positive cells per high power field (200×; n=10 fields per section). (E-G**)** The ratio of areas with positive expression of AhR, IRF1, HIF-1α in IHC. (H) The ratio of areas with positive expression of miR-142 in FISH. n = 6 per group. The data are shown as mean ± SD. *P < 0.05; **P < 0.01, as determined by one-way ANOVA (A-H).

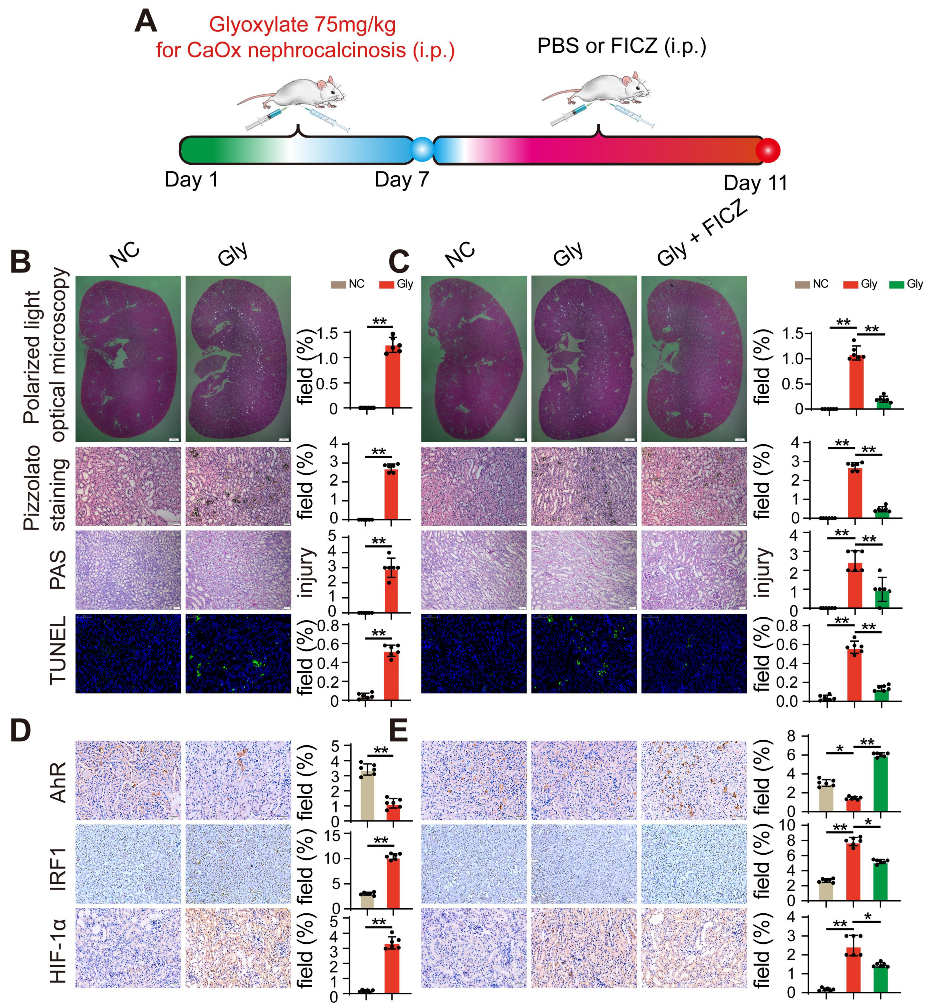

Figure S8. AhR activation suppressed the deposition of CaOx crystals and CaOx nephrocalcinosis induced kidney inflammatory injury. (A) Experimental overview. (B, C) Deposition of renal CaOx crystals in the corticomedullary junction of mice (n = 6) treated with glyoxylate (Gly) (75 mg/kg/d) and/or FICZ was analyzed via polarized light optical microscopy (20×, scale bar: 500 μm). Crystals in corticomedullary junction regions was further measured by Pizzolato staining (200×; scale bar: 20 μm). Kidney injury and necrosis were evaluated by PAS staining (200×; scale bar: 20 μm) and TUNEL staining (200×; scale bar: 100 μm) in kidney tissues, respectively. (D, E) IHC staining for AhR, IRF1, and HIF-1α in kidney tissues (200×; scale bar: 20 μm). n = 6 per group. The data are shown as mean ± SD. *P < 0.05; **P < 0.01, as determined by Student's t test (B, D) or one-way ANOVA (C, E).
